# Experimental procedures for studying microbial reactions under high hydrogen gas saturations in microcosms

**DOI:** 10.1016/j.mex.2025.103344

**Published:** 2025-05-10

**Authors:** Aidan Jaques, Mark Ireland, Cees van der Land, Reinhard Dirmeier, Nicole Williamson, Beate Christgen

**Affiliations:** a School of Natural and Environmental Sciences, Newcastle University, England, United Kingdom; b BP Biosciences Center, San Diego, USA

**Keywords:** Hydrogen, Microbial metabolism, Microcosm studies, Methanogenesis, Sulfate reducing bacteria, Acetogenesis, Microbiology studies under high saturations of hydrogen in headspace in microcosms

## Abstract

This methodology is proposed to investigate the response of microbial communities through analysis of headspace composition under high saturations of hydrogen. Changes in headspace composition will be related to specific communities and environmental conditions that will influence their response and result in changes in gases produced or potential changes in the liquid phase of microcosms pertaining to the hydrogen consumption rate through microbial metabolic processes. A step-by-step procedure is documented here.

• Methodology includes an easy setup utilising common laboratory equipment.
• The method showed minor appreciable loss of hydrogen from the microcosm setup/storage and the use of exetainers for gas measurements.
• Actively studied microbial hydrogen consumption across 18 days at 30 °C and 50 °C

This method is useful for the first instances in scientific studies towards understanding species or microbial communities found in environments with high percentages of hydrogen: underground hydrogen storage sites, hydrogen pipelines, and hydrogen leakage into subsurface soils.

## Graphical abstract

**Figure:**
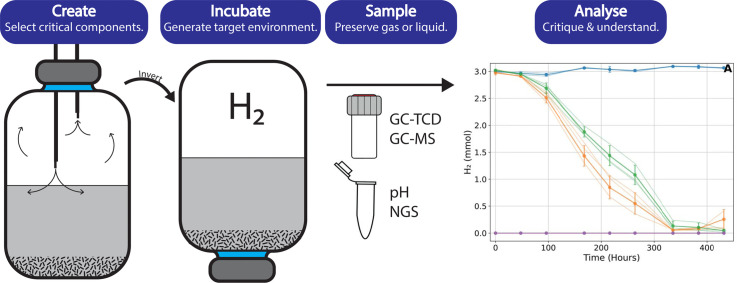

Specifications table

**Table:** 

Subject area:	Environmental Science
More specific subject area:	Hydrogenotrophic geomicrobiology
Name of your method:	Microbiology studies under high saturations of hydrogen in headspace in microcosms
Name and reference of original method:	Pitombo, Ramos et al. [1] “Methodology for soil respirometric assays: Step by step and guidelines to measure fluxes of trace gases using microcosms.” MethodsX**5**: 656–668
Resource availability:	Wheaton bottles (250 mL - borosilicate), crimp top seal Butyl rubber stoppers 20 mm aluminium crimp seals Crimp sealer 3 mL double-wadded borosilicate exetainers Hydrogen gas (50 %)/nitrogen gas (50 %) mix Microbial inocula
	Synthetic brine/environmental sample Duran bottles (1 L) Weighing scale Terumo agani needles (19G-25 G) Terumo agani syringe with luer lock (1mL-5 mL) Hamilton Syringe 1002/1710 SL Strippettes (25mL-50 mL) Gas Chromatograph – Thermal Conductivity Detector and Mass Spectrometer pH probe Fume hood Incubator

## Background

Microcosms are a useful, low cost, straightforward, and reproducible experimental method with applications in numerous fields of study including, soil systems [2], aquatic systems [3], terrestrial plants [4] and sediment [5] and significantly aid in understanding microbial processes in diverse environments [6]. Measuring microbial communities in situ is both costly and adds complexities due to the difficulty of isolating the variables that affect microbial processes. Using microcosms to study microbial communities under high concentrations of hydrogen simplifies the environmental conditions and allows for investigation into and control over these variables. With the increase of hydrogen in developing energy systems to aid Net Zero, microorganisms can utilise this hydrogen as part of their metabolism, which can have consequent effects on the system where they are found [7]. Understanding these processes is important for environmental management and monitoring if hydrogen leaks into the environment from pipes or underground storage sites. Recently, Dopffel, Mayers et al. [8] demonstrated a microcosm that represented conditions during underground hydrogen storage to provide experimental data describing hydrogen-related metabolic reactions in microorganisms. Our method is both important and novel, as it addresses the challenges of working with hydrogen to produce reliable and reproducible data due to its fugitive nature. This method effectively mitigates those difficulties. Subsurface conditions (i.e., temperature and salinity) can be replicated in a laboratory setting using multiple microcosms to study how these environmental factors influence microbial community hydrogen consumption. Our method goes through the setup using a hydrogen-rich headspace in microcosms with recommendations of amendments tailored to fit the user's requirements, while demonstrating a reproducible and reliable approach regardless of microbial origin. Here, we used a mixed inocula source from river sediments however, the method could utilise single or mixed cultures from other sources. In our setup, we can measure hydrogen levels over time and additional gaseous hydrogenotrophic metabolic products (i.e., CH_4_, CO_2_, H_2_S) of interest to the study. Individual chemical safety forms, i.e., UK-specific Biological agents/Control of Substances Hazardous to Health (BIO/COSHH) or Dangerous Substances and Explosive Atmospheres Regulations (DSEAR) or equivalent international laboratory health and safety standards, must be completed per laboratory to store hydrogen in these microcosms and incubators safely.

## Method details

### Media and microcosms

Inoculum was sourced from sediment sampled from the River Tyne, as it is known to be highly diverse in microorganisms and contains a high organic content with known halophilic, mesophilic, and thermophilic microorganisms [9,10]. The sample was from a riverbank in an intertidal brackish zone (54.964250, −1.683111). The top 20 cm of sediment was removed to access the dark grey/black anoxic portion of the sediment. This was immediately placed into 1 L sterile HDPE bottles up to the brim, sealed, and refrigerated at 4 °C until microcosm preparation. Exposure of the environmental sample to long durations of oxygen during collection may affect the obligate anaerobic microorganisms and distort the microbial community. Environmental samples should be collected quickly and close to the microcosm creation date/experiment start date. Environmental samples should be purged and stored with a nitrogen headspace for extended storage periods before the experiment setup, especially if the microbial community is anaerobic.

Media and culture conditions were selected to encourage microbial growth and consequent hydrogen consumption, with conditions optimised to reduce limiting elements and micronutrients. For this the Widdel and Bak [11] basal media and Widdel [12] media were used as the nutrient source for all experimental setups. The basal media and the nutrient source are specifically used for sulfate-reducing bacteria due to the availability of sulfur; however, all other elements, vitamin solutions, and trace elements are widely used by anaerobic microorganisms such as methanogens and acetogens [12]. Microcosms set up used to display the method, in triplicate, a microcosm containing inocula and sulfate, a microcosm containing inocula and no additional sulfate, a microcosm containing inocula and nitrogen, and a microcosm containing distilled water and no inocula.

The following solutions from Widdel and Bak [11] were used: basal media, trace element mixture, vitamin mixture, selenite tungstate solution, thiamine solution, vitamin B12 solution, bicarbonate mixture, and sulphide solution (Table 1). The brine media was deoxygenated with nitrogen for 30 min at 2 psi before microcosm setup.

**Table 1:** Make up of microcosms from Widdel and Bak [11].

Solution	Volume (ml)	Sterilisation Method
Basal stock media	957.5	Autoclaved
Trace element mixture	1	Autoclaved
Vitamin mixture	1	Filter (0.2 µm)
Selenite tungstate	1	Autoclaved
Thiamine solution	1	Filter (0.2 µm)
Bicarbonate solution	30	Autoclaved
Vitamin B12 solution	1	Filter (0.2 µm)
Sulfide solution	7.5	Autoclaved

### Media pH adjustment

Before media addition to microcosms, pH may be adjusted to fit the system that is being investigated or the microorganism/community for optimum growth. In this instance, the pH was adjusted in the Widdel and Bak basal media to pH 7, using NaOH/HCl.

### Setting up microcosms

**Fig. 1:**
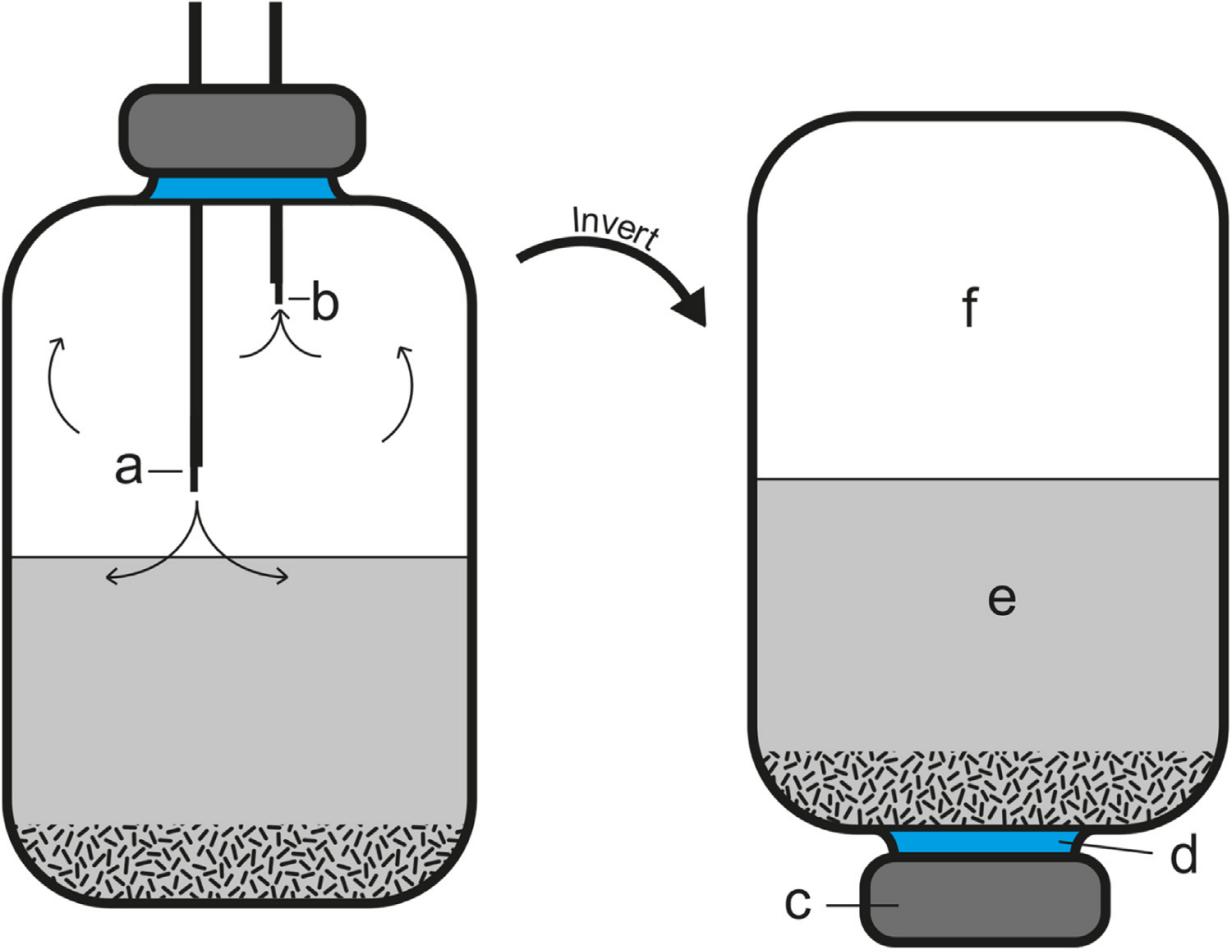
An illustration of a microcosm in a Wheaton crimp seal bottle during headspace flushing and after inverting for reduced hydrogen loss during incubation. (a) Injection gas needle, 19 G (1.1 × 50 mm), attached to gas canister of original headspace mix 50 % hydrogen and 50 % nitrogen; (b) Exit/venting needle, 21 G (0.8 × 38 mm), for flushed headspace to escape and avoid over pressurisation; (c) Aluminium crimp seal; (d) Butyl rubber stopper; (e) Basal media with additional micronutrients/vitamins and sourced inocula; (f) Headspace starting mix, 50 % hydrogen and 50 % nitrogen.

Incubations were performed in 250 mL borosilicate crimp seal Wheaton bottles (Fig. 1). Inocula was added to the Wheaton bottles at 5.0 g via sterile syringes. It is difficult to provide identical inocula from an environmental sample, so to limit mass differences in microorganisms from the inocula in Wheaton bottles, the sediment was mixed thoroughly to remove aggregated clumps and air pockets, added to the syringe, and injected into the microcosm. Subsequently, 100 mL of the basal media was added to the Wheaton bottle via strippettes and sealed with a butyl rubber stopper and crimp sealed with a 20 mm aluminium crimp seal. During this movement of basal media, a nitrogen line was bubbling through the basal media and microcosm to maintain anoxic conditions as much as possible. Microcosm headspace was then flushed with nitrogen for 5 min, followed by a mixture of 50 % hydrogen and 50 % nitrogen for 5 min at 3 psi using an inject needle and exit needle (19 G (1.1 × 50 mm) and 21 G (0.8 × 38 mm) Terumo Agani needles were used). Both needles were removed simultaneously upon reaching 5 min. To minimise the loss of hydrogen through the puncturing process, the microcosms were inverted to ensure the liquid phase was in contact with the stopper during incubation [8]. Microcosms were kept at 30 °C and 50 °C incubators for the duration of the 18-day experiment and were only taken out for sampling.

### Sampling

Before puncturing the rubber stopper, the needle and syringe were flushed three times with the hydrogen/nitrogen mix used to reduce the risk of oxygen entering the microcosm. A second running nitrogen line was also present over the head of the microcosm to be sampled at 2 psi. The needles used for sampling were 25 G (0.5 × 25 mm) Terumo Agani needles for sampling headspace and 22 G (0.8 × 38 mm) Terumo Agani needles for sampling the liquid phase. The larger aperture of the 22 G needle allows the removal of inocula for any later microbial analysis, and in this case, does not internally get clogged with sediment. Syringes used were 5 mL Terumo concentric luer-lock tip to withdraw gas directly from microcosms and additional gas-tight syringes, Hamilton 1002/1710 SL, for injection from exetainers into the Gas Chromatograph with Thermal Conductivity Detector (GC -TCD) and Gas Chromatography-Mass Spectrometer (GC–MS), respectively.

The sample volume of media taken from microcosms will depend on the size of the microcosm, the total volume of media, measuring frequency, and what is to be analysed, e.g., Gaseous H_2_, CO_2_, CH_4_, or liquid pH, sulphide [13], VFA’s, liquid chromatography. In this experiment, a 4.5 mL headspace sample was taken three times a week with purged needles and syringes (with 50 % H_2_/N_2_) and stored in the pre-evacuated exetainers. Liquid samples of 2 mL were taken once a week and kept in 5 mL PCR tubes to test for pH, excluding the final day of the experiment (Day 18) where a final liquid sample was taken to test the end pH, and a final community could be established (via 16S rRNA sequencing). The pH was recorded using a Mettler Toledo Micro pH electrode in triplicate following a triple-point calibration. To maintain internal pressure within microcosms, 4.5 mL of the original gas mix is reinjected into the microcosm. This additional hydrogen being reinjected is a known concentration and can be adjusted for in the final mmol calculation.

### Exetainers

The use of exetainers has the advantage of pausing a specific headspace composition in time from a microcosm and has been used previously in other studies [14]. In this setup, pre-evacuated 3 mL double-wadded borosilicate exetainers (from Labco) were used to ensure the highest sealing capability for storing hydrogen. Exetainers were pre-evacuated, so only the microcosm sample was stored, not an unknown atmospheric composition. Samples were stored for 2–5 days till GC analysis. Additionally, using these exetainers is a larger headspace sampling size, which may not be appropriate for smaller Wheaton bottles.

### Analytical analysis

In order to limit hydrogen loss through the exetainer septa, hydrogen was first analysed on an SRI 8610C gas chromatograph with Thermal Conductivity Detector (TCD). Before sample injection, the GC-TCD was baked out at 300 °C for 2.5 h to remove any residual contaminants from the column. Aliquots (1.5 mL) of gas from exetainers were sampled using a 2.5 mL gastight Hamilton 1002SL syringe and 26S Hamilton needle. Gases were injected via a 0.5 mL sample loop onto a 6 ft long packed molecular sieve 5 Å column at a constant pressure of 30 psi with nitrogen carrier gas, column temperature of 40 °C, and TCD temperature of 100 °C. After every nine samples run, a drift standard of 1.5 mL of 1 % H_2_/99 % N_2_ was run to correct for any drift. Each sample's peak area was manually integrated. Due to the larger thermal conductivity difference, nitrogen may be replaced as a carrier gas with argon. It may be appropriate for measuring hydrogen-generating microcosms in significantly lower quantities at the ppm level. In our experiment, only nitrogen was used.

Additional gas phase hydrogenotrophic metabolic products (CH_4_, CO_2_, H_2_S) were analysed on an Agilent 5977B Gas Chromatograph – Mass Spectrometer. This used helium gas as a carrier at 49.5mL/min and 6.181 psi with oven temperature at 35 °C and heat at 150 °C. Standards of 1 % CH_4_/CO_2_ 98 % N_2_ and 10 % CH_4_/CO_2_, 80 % N_2_ were run at 20 µL, 40 µL, 60 µL, 80 µL, and 100 µL, respectively prior to manual injection of 100 µL from exetainer samples every 2 min.

### Calculus

Peak areas generated by the GC-TCD were calibrated through two control standards of 1 % H_2_ and 50 % H_2_ with N_2_. A linear calibration curve was generated (Supplementary, Table 3, Fig. 4), and the relationship between peak area and volume (mL) could be determined using the slope-intercept equation. Gas concentrations could then be converted to units of mmoL using the ideal gas law. Peak areas generated by the GC–MS were automatically calculated by the GCMS software. Using the standards of 1 % CH_4_/CO_2_, a linear calibration curve was generated, and peak area converted into mmoL using the ideal gas law.

### Sensibility and quality control

**Fig. 2:**
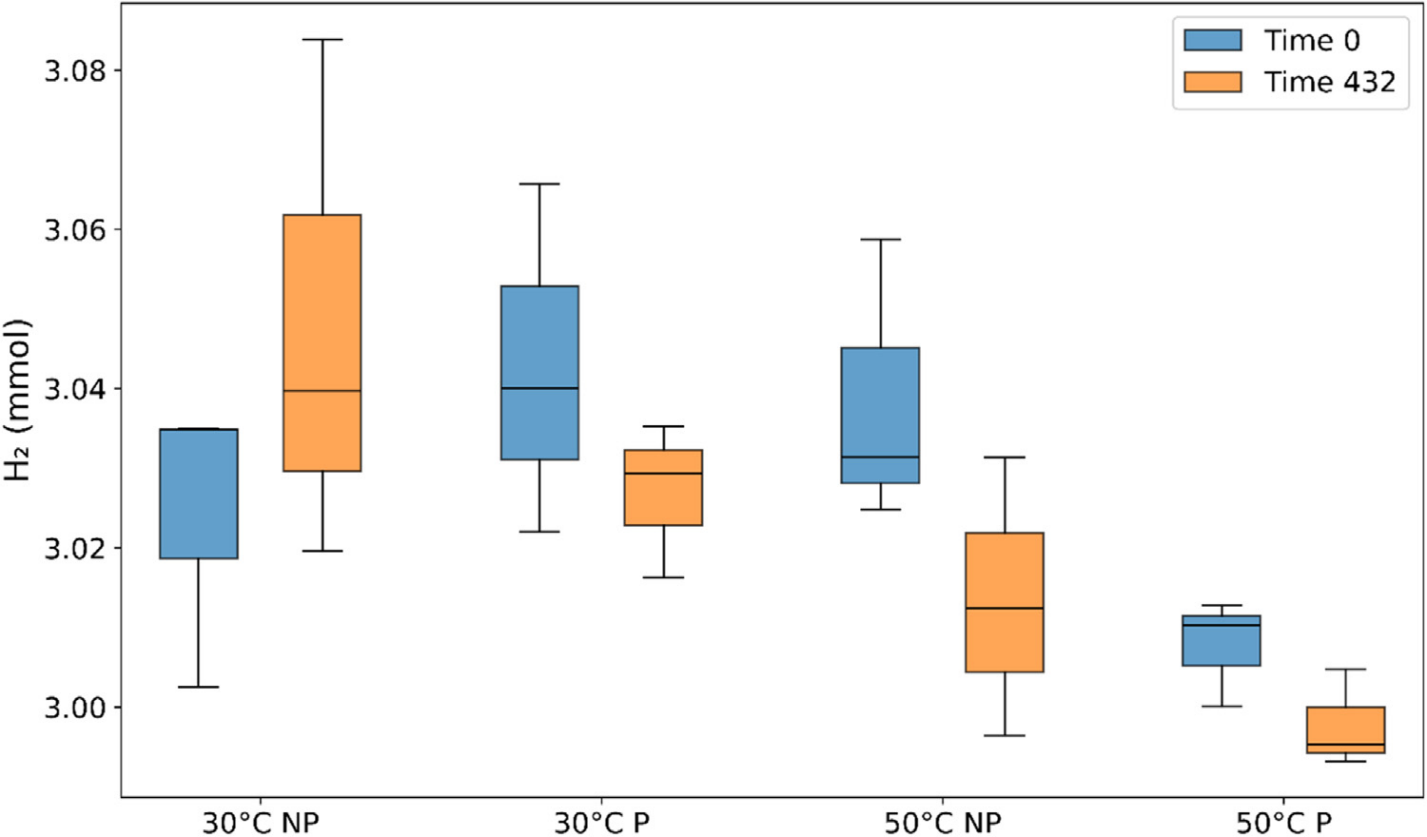
Hydrogen was tested from distilled water and different puncturing routines in microcosms. Time series at time point 0, immediately after microcosm creation, and time point end (day 18). NP = non-punctured rubber stoppers over time until the final sample day 18. *P* = punctured microcosm at the same sample period of the 18-day experiment.

Containment of hydrogen during incubation is an important factor, inevitably, there will be some minor hydrogen loss each time the rubber stopper is punctured during sampling. To ensure that any changes in hydrogen (and other gases) during the experiment can be attributed to processes within the microcosm, this loss through the rubber stopper needs to be understood. For this, two sets of triplicates of hydrogen with distilled water in Wheaton bottles with no microorganism source were left at 30 °C and 50 °C for 18 days of incubation (Fig. 2). One set of triplicates was without puncturing the rubber stopper after the headspace flushing, and the other was punctured during the experimental method's sampling period. Table 2, holds mmoL data of how much hydrogen was lost at each temperature and punctured/non-punctured condition. To quantify measurement accuracy, we calculated the coefficient of variation (CV) for all microcosm measurements, finding values between 0.16 % and 0.88 %, which indicates high measurement accuracy, Table 2. Additionally, calculated in the supplementary material (Supp, Table 3), is the CV for the gas standard measurements used for linear calibration (Supp, Fig. 4). There was less hydrogen loss from the 30 °C microcosms than the 50 °C microcosms. At 50 °C, minor hydrogen loss occurred in both the non-punctured and punctured microcosms. However, the results fall within the range of the TCD detection error limit. The largest overall loss of hydrogen was 0.8 % at 50 °C with the non-punctured microcosm. This discrepancy can be accounted for in each experiment's time length and puncturing routine. This hydrogen loss can be amended into the results by applying a correction factor to all experiments based on the observed loss in these controls, enhancing the overall accuracy of measured hydrogen consumption levels.

**Table 2:** Mean H_2_mmoL and CV from NP and P storage experiment.

Temperature ( °C)	Condition	Mean H_2_ (mmoL) T0	Mean H_2_ (mmoL) T432	CV 0	CV 432	STDev 0	STDev 432
30	NP	3.0240	3.0477	0.5052	0.8812	0.0152	0.0268
30	P	3.0426	3.0269	0.5893	0.2612	0.0179	0.0079
50	NP	3.0383	3.0134	0.4832	0.4733	0.0147	0.0142
50	P	3.0077	2.9977	0.1823	0.1672	0.0054	0.0050

NP = non-punctured microcosms. *P* = punctured microcosms. CV = Coefficient of variation. STDev = Standard deviation of H_2_ mmoL.

**Table 3:** CV stats for linear calibration.

Line	CV 1 %	CV 50 %	R^2^
1	0.76	0.09	1.000
2	0.41	0.14	1.000
3	0.84	0.09	1.000

The detection limit of this method, 0.1 % hydrogen (1000 ppm), is defined by the sensitivity of the TCD. A lower concentration of 0.01 % (100 ppm) hydrogen in nitrogen gas standard was also tested, but the peak area was poor and could not easily be distinguished. If detecting changes in very low concentrations of hydrogen, for instance, if investigating hydrogen production instead of consumption, the TCD program parameters would have to be adjusted. For instance, reducing the flow rate over the column loop or changing the carrier gas (i.e., argon instead of nitrogen). To observe changes in high concentrations of hydrogen, as in our experiment, the method using nitrogen as carrier gas and 30 psi was suitable.

The TCD could be used to analyse H_2_, CO_2,_ and CH_4_. Increasing the duration of the run, ramping temperature, and changing the psi pressure on the GC-TCD enables measurement of all three gases, albeit with a reduction in the sensitivity and an increase in time required. Therefore, we recommend using a standard GC–MS for additional gases where detection limits are more sensitive. The detection limits of gases on a GC–MS will also vary with machine and run parameters. In most instances, GC–MS detection limits will be readable to 0.1 % and lower.

A sufficiently sensitive probe for pH variations should be used and calibrated to the correct range and temperature conditions. In this experiment, the pH probe was calibrated with 4.01, 7, and 10 pH standards before readings, and technical triplicate readings were recorded for each microcosm sample. Recording pH should be done promptly upon sample collection, followed by storage within freezers to preserve microbial communities.

## Method validation

**Fig. 3:**
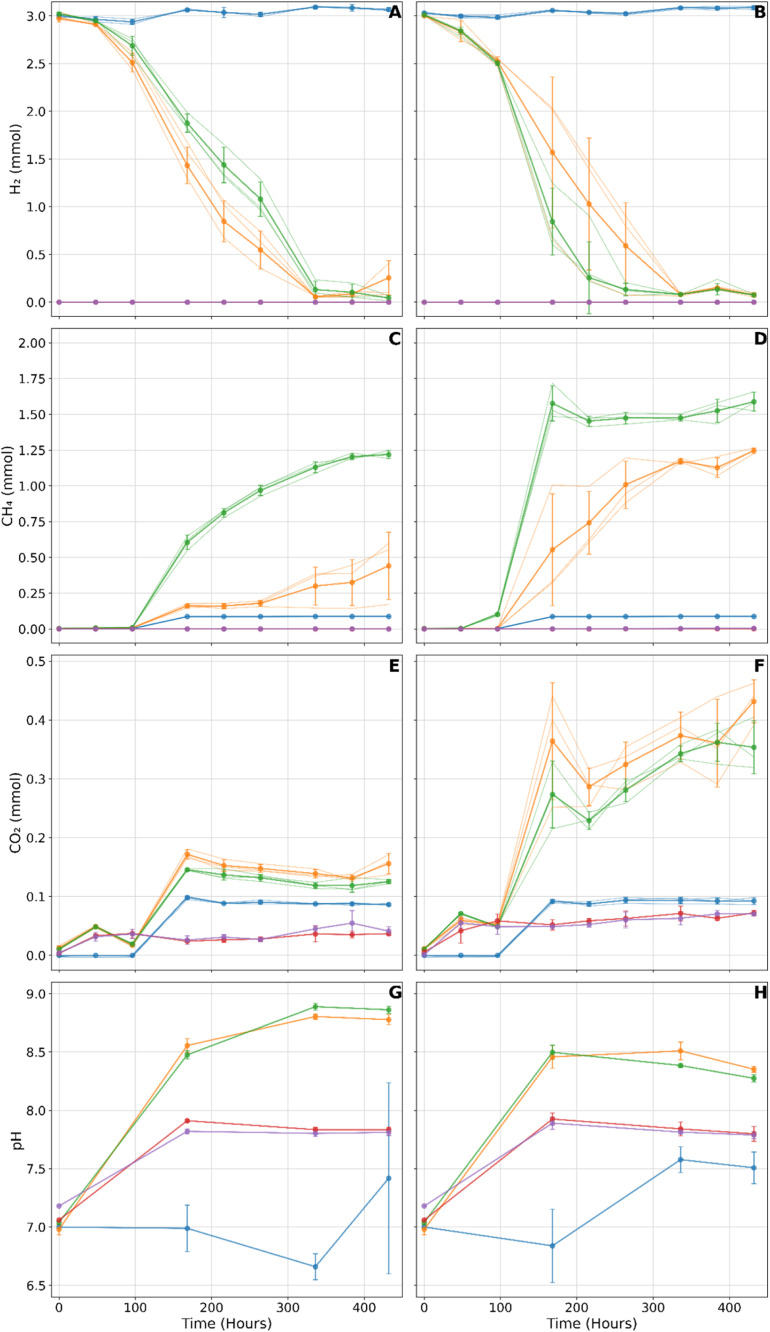
Blue Line = Negative control of distilled water. Orange line = Microcosms containing sulfate. Green line = Microcosms not containing sulfate. Red line = Microcosms containing nitrogen in headspace and sulfate. Purple line = Microcosms containing nitrogen in headspace and no sulfate. A) Recorded hydrogen in mmoL at 30 °C. B) Recorded hydrogen in mmoL at 50 °C. C) Methane in mmoL at 30 °C.D) Methane in mmoL at 50 °C. E) Carbon Dioxide in mmoL at 30 °C. F) Carbon dioxide in mmoL at 50 °C. G) pH across microcosms at 30 °C. H) pH across microcosms at 50 °C.

Example results from the method described are shown in Fig. 3, presenting replicate microcosms and the overall mean. The results from the negative controls demonstrate that hydrogen is contained within the microcosms with 0.615 % loss over the 3 weeks. In contrast, inoculated hydrogen microcosms demonstrate an increased hydrogen consumption of nearly 100 % in both sulfate and non-sulfate bearing microcosms at 30 °C and 50 °C (Fig. 3A, 2B) by experiment end. Measurements of the additional gases produced during the experiments provide further evidence of the active utilisation of hydrogen by the microbial community (Fig. 3C-F). Data variability is evident in some microcosms due to different compositions/relative abundance of hydrogenotrophic microorganisms even in replicate microcosms, as observed in sulfate-bearing triplicates (Fig. 3B-D), and wider erroneous pH readings in the control distilled water microcosms Fig. 3G Here, one sulfate-bearing microcosm exhibits enhanced hydrogen consumption rates and comparably higher instances of methane production, leading to wider error bars. Instances like this could be removed and classified as outliers or investigated further. This variability could potentially be reduced using pure cultures or possibly accounted for by conducting cell counts per microcosm beforehand. Triplicate microcosms are standard practice for laboratory experiments and enable the assessment of the reproducibility and intrinsic variability in natural systems of the experimental setup. Creating more microcosms, such as quintuplicates or more, could help exclude potential outliers from the final results. For the distilled water microcosm controls, it is difficult to measure pH in a system where there is a lack of hydrogen ions, which the pH meter records, hence the variability, apparent drift, and larger error bars in the pH controls.

**Fig. 4:**
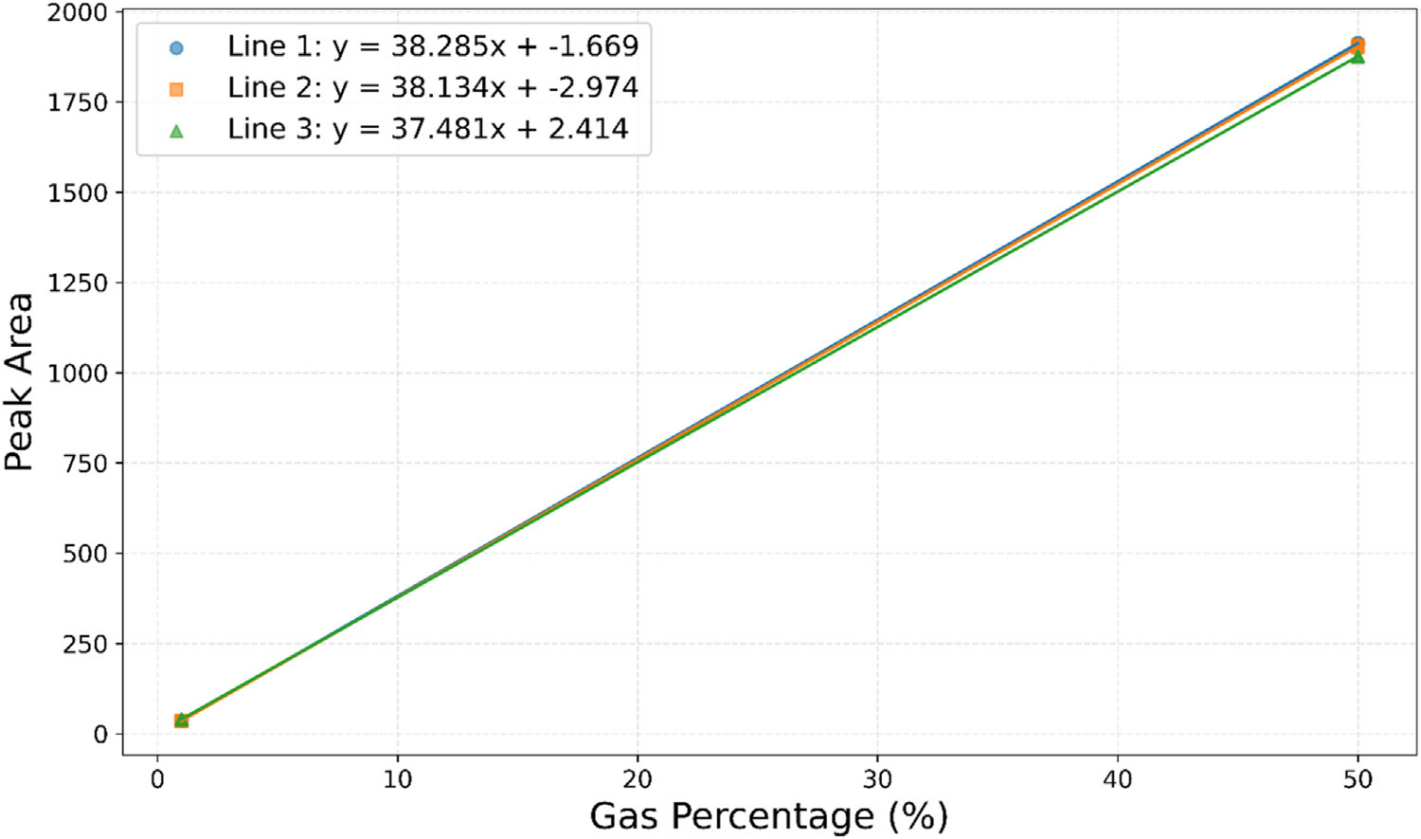
Standard linear line for H_2_ % of 1 % and 50 %.

Sampling frequency depends on the planned experiment length, intensity of microbial activity, and the environment being researched. In some instances, daily sampling of headspace may be required, and in others, maybe monthly sampling, depending on microbial growth rate and the environmental conditions used in the microcosms. In this experiment, recording every other day, excluding weekends, was sufficient to observe an active community utilising hydrogen.

Storing for longer periods or puncturing the septa on the exetainer for any further analysis may result in the loss of hydrogen from the exetainer. Hydrogen loss from storage in exetainers for the 2–5 days before running on the TCD was negligible and did not show changes between 2 or 5 days of storage. Exetainers were tested by storing 4.5 mL of 50 % H_2_/N_2_ gas for 21 days and running the samples on the TCD; minor hydrogen loss was observed (Appendix Table 4). Other gases (CH_4_/CO_2_) also demonstrated no loss from the exetainer and are better suited to longer-term storage. Readings from both GCs were left unaffected by this use of exetainer storage.

**Table 4:** Exetainer storage at 1 day and 21 days compared to the fresh standard of 50 % from a gas bag.

Exetainer	H_2_ mmoL – 1 day	H_2_ mmoL −21 days	H_2_ mmoL - fresh	H_2_ % lost – Day 1 – Day 21	H_2_ % lost – bag to day 21
1	3.0307	3.0245	3.1107	0.2046	2.7712
2	3.0239	3.021	3.1145	0.0959	3.0016
3	3.0501	3.0238	3.1278	0.8623	3.3253

## Limitations

This experimental approach of studying community microbial hydrogen consumption uses microcosms, which offer a simple setup requiring minimal skill. The method is reproducible, scalable to different environmental conditions and microbial sources, cost-effective, and safer than conducting full-scale field studies. These advantages make it a practical tool for assessing microbial risks associated with hydrogen-rich environments. There are several notable limitations to be aware of and consider when utilising this method to interpret results or design follow-up experiments.

One of the main limitations of this experimental method is researching microcosms with hydrogen ratios exceeding 50 % in the headspace. Headspace pressure is maintained close to atmospheric balance to minimize exchange with atmospheric gases during sampling and to allow easy withdrawal of gas samples. The extracted gas will contain a proportionally higher amount of nitrogen, and thus, a quantitative measurement can be achieved via the GC-TCD. Other methods should be investigated if research/experiments look into >50 % hydrogen in the headspace. Additionally, nitrogen is part of the nitrogen fixation pathway, which includes hydrogen in its metabolic pathway. Altering or removing this nitrogen gas, such as replacing it with argon, will allow a nitrogen fixing community to consume minimal hydrogen. Furthermore, working with 50 % hydrogen is safer than 100 % hydrogen. While gas containing 5 % hydrogen is considered flammable, the inert gas (nitrogen) in the microcosm bottle can limit an explosion radius [15].

Microcosm research in Wheaton bottles is limited by the pressures at which it can be conducted, with many being conducted at atmospheric pressures, with any internal changes from gas expansion under the microcosm bottles' incubation temperatures. Pressures exceeding 1.5 atmospheres are generally unsuitable for Wheaton bottles, and other experimental methods have to be investigated for high-pressure systems. However, pressure itself on microbial growth rate does not have a major limitation, where many piezophilic/mesophilic/thermophilic microorganisms are adapted to increased pressure conditions [16].

Reducing the reliance on exetainers will depend on GC availability or column size on the TCD. Utilising a smaller sample loop on the GC-TCD would allow for directly injecting gas from the microcosm onto the GC-TCD. The same is true for direct injection from microcosms to GC-MS. The only downside to this is puncturing the butyl rubber stopper multiple times, which could potentially increase the hydrogen loss rate through the stopper over time.

Rubber stoppers are expected to degrade over time depending on the chemical makeup of the stopper, incubation under higher temperatures, the aperture of needles puncturing the stopper, and sampling frequency. It is advisable to fully understand the sampling frequency of the experiment beforehand and select the appropriate needles to minimize hydrogen loss through the rubber stopper.

Lastly, this experimental method suits systems that have a sufficient liquid phase to provide a barrier between the headspace and the rubber stopper. Dry systems, where there is no liquid contact between the headspace and rubber stopper, may be unsuitable for long-term experiments, as hydrogen has increased chances of escaping due to direct headspace contact with the rubber stopper.

Concluding remarks:

• Microcosms can be set up using material/equipment widely available in standard academic or commercial laboratories.
• The method is easily repeatable and adaptable to different conditions/environments/microorganisms.
• Changes in the headspace gas composition, in this case, hydrogen consumption, can be calculated by measuring the headspace over time. Coupled with additional gas measurements from active communities, the dominant hydrogen consumer might be established depending on microcosm conditions.

Scheduling timed microcosm destruction or subsampling supernatant for further analysis can be used to study community differences over time under hydrogen through 16S rRNA analysis.

## Ethics statements

N/A.

## CRediT authorship contribution statement

**Aidan Jaques:** Conceptualization, Methodology, Validation, Formal analysis, Investigation, Writing – original draft, Visualization. **Mark Ireland:** Conceptualization, Writing – review & editing, Supervision, Project administration, Funding acquisition. **Cees van der Land:** Writing – review & editing, Supervision. **Reinhard Dirmeier:** Writing – review & editing, Supervision, Project administration, Funding acquisition. **Nicole Williamson:** Writing – review & editing, Supervision, Project administration. **Beate Christgen:** Writing – review & editing.

## Declaration of competing interest

The authors declare that they have no known competing financial interests or personal relationships that could have appeared to influence the work reported in this paper.

## Data Availability

Data will be made available on request.

## Appendix Supplementary materials

Image, application 1
