# Supplementary material for: Experimental procedures for studying microbial reactions under high hydrogen gas saturations in microcosms
Source: MethodsX. 2025 May 10;14:103344. doi: 10.1016/j.mex.2025.103344 (PMC12139486; doi:10.1016/j.mex.2025.103344)
Supplement: Supplementary file 1 [file mmc1.docx]

Supplementary Material: Experimental procedures for studying microbial reactions under high hydrogen gas saturations in microcosms*.*


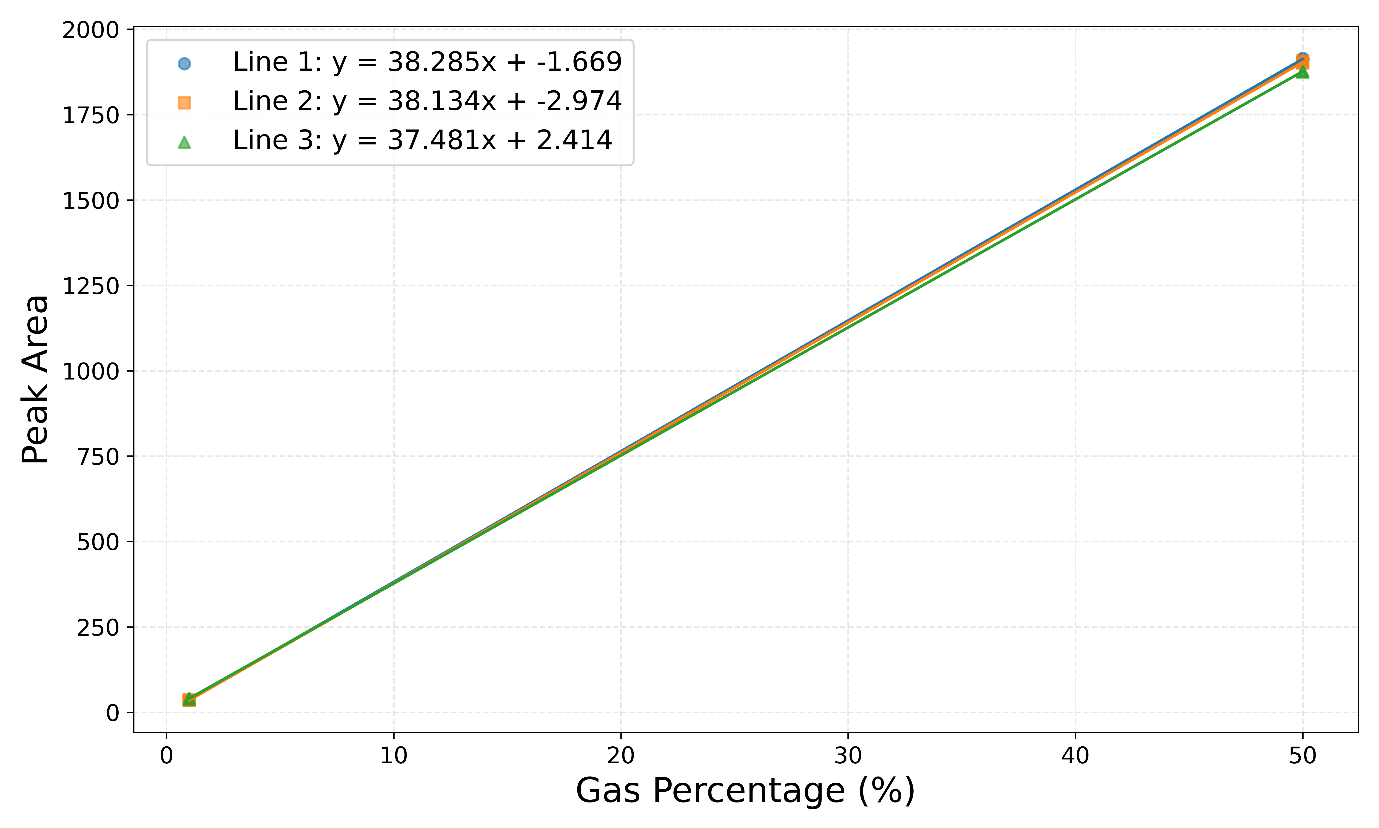


Figure 1: Standard linear line for H_2_% of 1% and 50%.

Table 1: CV stats for linear calibration.

| Line | CV 1% | CV 50% | R^2^ |
| --- | --- | --- | --- |
| 1 | 0.76 | 0.09 | 1.000 |
| 2 | 0.41 | 0.14 | 1.000 |
| 3 | 0.84 | 0.09 | 1.000 |

Table 2: Exetainer storage at 1 day and 21 days compared to the fresh standard of 50% from a gas bag.

| Exetainer | H_2_ mmoL – 1 day | H_2_ mmoL -21 days | H_2_ mmoL - fresh | H_2_ % lost – Day 1 – Day 21 | H_2_ % lost – bag to day 21 |
| --- | --- | --- | --- | --- | --- |
| 1 | 3.0307 | 3.0245 | 3.1107 | 0.2046 | 2.7712 |
| 2 | 3.0239 | 3.021 | 3.1145 | 0.0959 | 3.0016 |
| 3 | 3.0501 | 3.0238 | 3.1278 | 0.8623 | 3.3253 |
